# Pigtail catheter versus open surgical drainage in liver abscess management

**DOI:** 10.6026/973206300210414

**Published:** 2025-03-31

**Authors:** Mahesh Kinikar, Nitin R. Nangare, Hemchandra V. Nerlekar

**Affiliations:** 1 Department of Surgery, Krishna Institute of Medical Sciences, Krishna Vishwa Vidyapeeth (Deemed To Be University), Karad, Maharashtra, India

**Keywords:** Open surgical drainage, pigtail catheter, post-hospital stay, post-operative pain, post-operative time

## Abstract

Individualized treatment programs for liver abscesses are essential. Therefore it is of interest to compare and evaluate pigtail-catheter with open surgical drainage in liver abscesses. Hence, a total of 126 patients were divided randomly into 2 groups with 63 each open surgical drainage group and pigtail-catheter drainage group. We found that statistically significant difference was seen between the 2 groups for various variables. Moreover, pigtail-catheter drainage showed comparatively more effective results than open surgical drainage.

## Background:

In the human body, the liver is an organ that is both essential and important. This organ, which is situated at the distal end of the portal circulation, is susceptible to a number of systemic illnesses, including infections caused by bacteria, viruses and parasites [1]. Liver abscesses have been detected ever since Hippocrates, who proposed that the kind of fluid that is contained inside the abscess cavity may have an effect on the prognosis of a patient. Liver abscesse is infectious lesions that take up space in the liver and are space-occupying [2]. The most common forms of abscesses are those that are pyogenic and amoebic in nature. Pyogenic liver abscesses are a rare condition that can be fatal. The severity of the condition depends on the patient's other health problems and where the infection came from. Amoebic AL is widespread in tropical regions, notably in areas where "Entamoeba histolytica" is abundant [3]. A study has shown that, individuals (typically young men) who have weakened cell-mediated immune systems are more prevalent in developing these abscesses. Over the course of the past quarter of a century, the treatment of P hepatic abscesses and amoebic liver abscesses has seen significant advancement. Death rates have decreased by 5-30% as a result of this [4]. Studies have shown that, a liver abscess is a localized accumulation of pus that grows inside the parenchyma of the liver. This condition often develops as a result of an injury or an infection. We can attribute this condition to the spread of either bacterial, fungal, or parasitic infections via the portal circulation [5, 6]. As the most common type of liver abscesses according to many studies, AHA are caused by *klebsielliver* abscesses (K.) *pneumoniae* and Escherichia *(E.) coli*, which have been named as the main organisms that cause them. About 20% of liver abscesses is thought to be cryptogenic, which means that there is no clear cause [7, 8-9]. Reports indicate that the incidence of liver abscesses in Western nations ranges from 1.0 to 3.6 cases per 100,000 persons [10]. On the other hand, in Asian countries, this ratio may reach as high as 17 cases per 100,000 individuals within the same population. With survival rates ranging from 15% to 19%, a liver abscesses presents a substantial danger [11, 12-13]. On the other hand, the death rates from liver abscesses have gone down a lot thanks to advances in minimally invasive treatments and better ways to find them early [11]. Studies have shown that, liver abscesses drainage may be performed through surgical methods, which include open or laparoscopic procedures, or via pigtail catheterization, utilizing computed tomography or ultrasound guidance for precision [8, 14]. Numerous studies indicate that pigtail drainage is preferred over surgical drainage for various reasons [15, 16-17]. Because of this, pigtail image-guided drainage is the main way to treat problems that don't need surgery right away, like peritonitis. Surgical drainage could provide advantages for extensive, multi-loculated abscesses, particularly in cases associated with biliary disease [15]. Therefore, it is of interest to compare pigtail catheter drainage versus open surgical drainage in Liver abscesses.

## Materials and Methods:

The current comparative prospective type of study was conducted in the department of general surgery, KIMS, Karad with 126 patients in total from March 2022 to September 2024. They were further divided into 2 groups with 63 each *i.e.* open surgical drainage group and pigtail-catheter drainage group. After admission in the hospital, necessary particulars regarding the age, sex of the patients were recorded to evaluate post-op complication, pain, hospital stay, operative time and total cost.

## Inclusion criteria:

[1] Uncomplicated liver abscesses

[2] Age between 18 to 70 years

[3] Abscess size greater than 5 cm without any complication

## Exclusion criteria:

[1] Abscess size <5cm

[2] Multiple abscess cavities

[3] Sign and symptoms of peritonitis

[4] Acute abdominal emergency

## Results:

Table 1 shows that, age of patients Group A was 60.032±14.318 years and Group B was 59.635±14.428. It was statistically insignificant as the p value was 0.877. Table 2 shows that, majority of the patients 42(66.67%) in group A and 43 (63.02%) in Group B. Thus showed not-significant difference as the p value was 0.237. Table 3 shows that, post-operative time Group A was 15±3.810 and Group B was 60±11.36. It was statistically significant as the p value was <0.0001 respectively. Table 4 shows that, P-HS Group A was 5.063±1.401 and Group B was 7.016±1.571. It was statistically significant as the p value was <0.0001 respectively. Table 5 shows that, Post-operative time, Group A mean and standard deviation was 1.206±0.626 and Group B was 1.921±0.848. It was statistically significant as the p value was <0.0001 respectively. Table 6 shows that, cost, group A was 1500±116.3975 and group B was 5015.873±861.2081. It was significantly correlated as the p value was <0.0001 respectively. Table 7 shows that, 1(1.59%) Wound Infection of pigtail patients with a liver abscesses was present. Table 8 shows that, 6(9.52%) pigtail blockage of pigtail patients with a liver abscesses was present. Table 9 shows that, 3(4.76%) pigtail dislodgement of pigtail patients with a liver abscesses was present. Table 10 shows that, 3(4.76%) Fistuliver abscesses Formation of pigtail patients with a liver abscesses was present. Table 11 shows that, 4(6.35%) post drainage peritonitis of open patients with a liver abscesses was present. Table 12 shows that, 8(12.70%) bleeding of open patients with a liver abscesses was present. Table 13 shows that, 7 (11.11%) wound infection of open patients with a liver abscesses was present.

## Discussion:

The incidence of draining liver abscesses has remained consistent since prior to the mid-20th century. Liver abscesses represent the most common extra-intestinal infection, occurring in 3-9% of patients. Research indicates a male-to-female ratio of approximately 2:1. The predominant age range for the occurrence of these disorders is between 40 and 60 years [15, 17]. Studies have shown that, liver abscesses represent the most prevalent form of intestinal infection [18, 19]. Conventional management strategies for liver abscesses encompass the utilization of pigtail catheter drainage. In specific instances, such as burst abscesses and multi loculated abscesses containing viscid pus, surgical evaluation is warranted; however, the literature indicates that only a limited number of these cases were examined [19, 20- 21]. Despite the fact that open surgical drainage is still necessary for the care of difficult liver abscesses cases, *percutaneous transhepatic drainage* has emerged as a preferred first method for a significant number of patients. These techniques are especially good for treating moderate to small abscesses that can be reached through the skin because they are minimally invasive have lower rates of complications and work just as well. When deciding whether to utilize *percutaneous transhepatic drainage* or open surgical drainage, it is important to take into account a number of factors, including the size and location of the abscess, the general condition of the patient and the level of expertise of the healthcare team that is engaged. When compared to open surgical drainage, *percutaneous transhepatic drainage* is a safer, more successful and more patient-centered approach for the therapy of liver abscesses. *percutaneous transhepatic drainage* encompasses a significant population of patients. The *percutaneous transhepatic drainage* technique offers a number of benefits, including the fact that it is a minimally invasive operation that may be carried out without the need of performing general anesthesia. Our study revealed a male preponderance, consistent with the findings of several previous studies [22, 23]. Local symptoms were used to show that the patients were getting better and there were differences in leucocytosis between the two groups [23, 24, 25, 26-27]. A study showed that, the conventional treatment for liver abscess is percutaneous catheter drainage, and is both safe and efficient. It leads to early symptom alleviation and quicker abscess cavity clearance. Surgery is an option for liver abscess drainage with concurrent intra-abdominal pathology, multi loculated abscess with biliary communication and failure of percutaneous drainage. Percutaneous catheter drainage also has low morbidity and a good success rate, allowing it to be used as first line management in liquefied moderate sized abscesses [28]. Another study reported that, the percutaneous catheter drainage is regarded as the standard treatment of choice and is a safe and effective method for managing liver abscesses. The intervention leads to prompt alleviation of symptoms and expedited closure of the abscess cavity. Percutaneous catheter drainage demonstrates low morbidity and a favorable success rate, making it a viable option for first-line management of liquefied moderate-sized abscesses. But surgery is still an option for draining liver abscesses when there is other disease going on inside the abdomen, when there are multiple abscesses that connect to bile ducts, or when percutaneous drainage has not worked [29].

## Conclusion:

Pigtail-catheter drainage is a more effective option than open surgical drainage as it offers a lot of wonderful advantages, including reduction in pain, length of hospital stays, problems and cost for treating Liver abscesses. This makes patients feel more comfortable with improved rehabilitation and it allows healthcare resources to function more efficiently. Thus, pigtail-catheter drainage is a preferred procedure for treating liver abscesses.

## Figures and Tables

**Table 1:** Age distribution

**Age**	**Group(A)**	**Group(B)**	**t-value**	**p-value**
Mean	60.03175	59.63492	0.55	0.877
SD	14.31779	14.42813		

**Table 2:** Gender distribution

**Gender**	**Group(A)**	**Group(B)**	**chi-square value**	**p-value**
M	42(66.67%)	43(68.02%)		
F	21(33.33%)	20(31.70%)	1.4	0.237

**Table 3:** Post OP. Time

**Post OP. Time (PP-T)**	**Group(A)**	**Group(B)**	**t-value**	**p-value**
Mean	15	60		
SD	3.810004	11.35924	29.81	p<0.0001

**Table 4:** Post of Hospital stay

**Post of Hospital stay (P-HS)**	**Group(A)**	**Group(B)**	**t-value**	**p-value**
Mean	5.063492	7.015873		
SD	1.401301	1.570823	7.36	p<0.0001

**Table 5:** Post OP. Pain

**Post OP. Pain(PP-P)**	**Group(A)**	**Group(B)**	**t-value**	**p-value**
Mean	1.206349	1.920635		
SD	0.626272	0.848178	5.378	p<0.0001

**Table 6:** Cost

**Cost**	**Group(A)**	**Group(B)**	**t-value**	**p-value**
Mean	1500	5015.873		
SD	116.3975	861.2081	39.42	P<0.0001

**Table 7:** Pigtail

	**Pigtail**	
**Wound Infection (WI)**	**Present**	**Absent**
	1	62

**Table 8:** Pigtail Blockage

	**Pigtail**	
**Pigtail Blockage (PT-BK)**	**Present**	**Absent**
	6	57

**Table 9:** Pigtail Dislodgement

	**Pigtail**	
**Pigtail Dislodgement (PT-DLG)**	**Present**	**Absent**
	3	60

**Table 10:** Fistuliver abscesses Formation

	**Pigtail**	
**Fistuliver abscesses Formation (FT-FM)**	**Present**	**Absent**
	3	60

**Table 11:** Post drainage peritonitis

	**Open**	
**Post drainage peritonitis (PD-PT)**	**Present**	**Absent**
	4	59

**Table 12:** Bleeding

	**Open**	
**Bleeding**	**Present**	**Absent**
	8	55

**Table 13:** Wound Infection

	**Open**	
**Wound Infection**	**Present**	**Absent**
	7	56
